# Development and Bone Regeneration Capacity of Premixed Magnesium Phosphate Cement Pastes

**DOI:** 10.3390/ma12132119

**Published:** 2019-07-01

**Authors:** Andrea Ewald, Dorothea Kreczy, Theresa Brückner, Uwe Gbureck, Melanie Bengel, Andreas Hoess, Berthold Nies, Julia Bator, Uwe Klammert, Andreas Fuchs

**Affiliations:** 1 Department for Functional Materials in Medicine and Dentistry, University Hospital Würzburg, Pleicherwall 2, 97070 Würzburg, Germany; 2 INNOTERE GmbH, Meissner Strasse 191, 01445 Radebeul, Germany; 3 Department of Oral & Maxillofacial Plastic Surgery, University Hospital Würzburg, Pleicherwall 2, 97070 Würzburg, Germany

**Keywords:** magnesium phosphate cement, prefabricated paste, implantation

## Abstract

Magnesium phosphate cements (MPC) have been demonstrated to have a superior bone regeneration capacity due to their good solubility under in vivo conditions. While in the past only aqueous MPC pastes have been applied, the current study describes the fabrication and in vitro/in vivo testing of an oil-based calcium doped magnesium phosphate (CaMgP) cement paste. Premixed oil-based pastes with CaMgP chemistry combine the advantages of conventional MPC such as high mechanical strength and good resorbability with a prolonged shelf-life and an easier clinical handling. The pastes set in an aqueous environment and predominantly form struvite and achieve a compressive strength of ~8–10 MPa after setting. The implantation into a drill-hole defect at the distal femoral condyle of New Zealand white rabbits over a course of 6 and 12 weeks demonstrated good biocompatibility of the materials without the formation of soft connective tissue or any signs of inflammation. In contrast to a hydroxyapatite forming reference paste, the premixed CaMgP pastes showed subsequent degradation and bony regeneration. The CaMgP cement pastes presented herein are promising bone replacement materials with excellent material properties for an improved and facilitated clinical application.

## 1. Introduction

Magnesium phosphate minerals such as struvite (MgNH_4_PO_4_·6H_2_O) [1] or newberyite (MgHPO_4_·3H_2_O) are currently discussed to be suitable alternatives to calcium phosphate phases for application as bone replacement materials [2]. This assumption is based on the higher solubility and biocompatibility of MgP minerals under in vivo conditions [3], which should lead to faster resorption and bone remodeling stimulated by released magnesium ions [4]. This behavior was proofed in a number of animal studies with models ranging from small defects in rabbits [5,6] up to partial load-bearing tibial defects in sheep [7]. Additionally, magnesium phosphate cements (MPC) turned out to show very good biomechanical features, which are even better than clinically well-established calcium phosphate bone fillers [8]. Since most of the implanted magnesium phosphate minerals are hydrated species, their processing and in vivo application usually follows a cementitious route, in which the starting minerals are mixed with an aqueous solution to form a paste. The latter shows a cementitious reaction due to the lower solubility of cement raw materials and the setting product and finally leads to the formation of a mechanically stable implant. For an overview about magnesium phosphate minerals and cement, the reader is referred to some recently published review articles describing in detail cement chemistry and biological testing [2,4]. 

Such MPC have high mechanical strength, but also a fast setting reaction limiting the processing time by a surgeon. Here, cement application in form of a premixed paste similar to approaches in calcium phosphate cement chemistry would be desirable to simplify application and to prolong the processing window for the surgeon [9,10,11,12]. Such premixed pastes are usually obtained by mixing the dry cement powder (including additives such as setting regulators) in an inert and water-miscible liquid like glycerol [11], polyethylenglycol [13], or oil/surfactant mixtures [9,14]. The obtained pastes have a long shelf-life in the absence of humidity and setting is initiated either after coming into contact with physiological fluids by diffusive water exchange or by actively mixing the pastes with an aqueous solution during the injection process. The latter is preferred for multicomponent cement systems to separate active cement components during storage for an improved cement paste stability and shelf-life.

This study hypothesizes that MPC powders can be transferred into premixed cement pastes by similar techniques as recently applied for calcium phosphate cements. For this purpose, the cement powders were suspended in a mixture of cytocompatible oil and surfactants leading to a long-term stable and injectable paste. Since previous studies have revealed that a partial substitution of the magnesium phosphate raw powder by calcium ions may have a further beneficial biological effect [15], two types of raw material with the general formula Ca_x_Mg_(3-x)_(PO_4_)_2_ (with x = 0.25 and 0.75) have been used. Setting of such cement pastes was obtained by actively mixing with an aqueous potassium phosphate solution as second reactant during injection with a commercially available static mixing device. In a first approach, the pastes were characterized regarding their setting behavior, mechanical properties, porosity and ion release into physiological medium. For analyzing the bone ingrowth of the cement pastes, a rabbit model well established for bony implant studies was used. Advantages of the rabbit model are the short lifespan of the animals and therefore early entrance into the adult state compared to larger animals. Additionally, the bone remodeling is more complex than in rodents, resembling the processes in human patients. According to these conditions and due to the demands of the animal welfare (3R: Replacement, reduction, and refinement) the model is frequently used for screening suitable materials before testing in a large animal model [16,17]. Therefore, the pastes were injected into femoral defects of New Zealand white rabbits and their bone regeneration capacity was evaluated by histological analyses after 6 and 12 weeks post implantation. As a control group, a commercially available hydroxyapatite (HA) forming premixed cement paste (INNOTERE Paste-CPC) was used.

## 2. Materials and Methods 

### 2.1. Cement Paste Preparation

Ca_0.25_Mg_2.75_(PO_4_)_2_ and Ca_0.75_Mg_2.25_(PO_4_)_2_ as cement raw materials were synthesized by sintering mixtures of MgHPO_4_·3H_2_O (Sigma-Aldrich, München, Germany), CaHPO_4_ (Baker, Schwerte, Germany), CaCO_3_ (Merck, Darmstadt, Germany) and Mg(OH)_2_ (VWR, Darmstadt, Germany) in appropriate stoichiometric ratios at 1100 °C for 5 h. The sintered cakes were manually crushed, sieved <125 µm and ground dry for 1 h in a ball mill (Retsch PM400, Retsch GmbH, Idar-Oberstein, Germany). The CaMgP raw powders were then mixed with finely ground (NH_4_)_2_HPO_4_ as reaction partner. The amount of the latter was equal to the amount in a powder-liquid-approach using 3.5 M (NH_4_)_2_HPO_4_ solution and a powder to liquid ratio of 3 to 1. Prefabricated cement pastes were then obtained by mixing the powder components with an oil-surfactant mixture in the ratio of 5.25:1 as described elsewhere [8]. During the individual experiments the CaMgP cement pastes were applied using a 4:1 double chamber cartridge system (Medmix, Risch-Rotkreuz, Switzerland) with a connected static mixer. For this, the cement pastes were transferred into the larger container of the cartridges, whereas the smaller container was filled with 0.5 M dipotassium hydrogenphosphate solution acting as reaction retarder and simultaneously providing water to initiate the cement reaction. 

As reference material a commercially available hydroxyapatite forming premixed cement paste (INNOTERE Paste-CPC, INNOTERE GmbH, Radebeul, Germany) was used. The material was transferred into the double chamber cartridges as described above, whereas saline solution was used as the liquid component. 

Finally, the filled cartridges were sterilized by γ-irradiation at 25 kGy to ensure sterility. 

### 2.2. Material Characterization 

The pastes were injected into silicon rubber molds with a size of 12 × 6 × 6 mm and hardened for 1 h at 37 °C in a water bath. Afterwards, the cement samples were removed from the molds and stored in phosphate buffered saline solution (PBS) for 30 d at 37 °C while changing the solution every second day. The mechanical properties of the set cements were determined under axial compression of the cuboid samples using a universal static mechanical testing machine (Zwick, Ulm, Germany) at a cross-head speed of 1 mm/min. The porosity was calculated by comparing the wet and dry weights of the samples after 2, 6, 12, 18, 24 and 30 d storage in PBS solution according to

P = (M_wet_ – M_dry_) / (d_PBS_ * V_sample_) (1)

where M_wet_ and M_dry_ are the weights of a sample after immersion in PBS and after drying for 24 h at 37 °C, d_PBS_ is the density of PBS and V_sample_ is the volume of the sample.

The ionic composition of the storage media was measured by inductively coupled plasma mass spectroscopy (ICP-MS, Varian, Darmstadt, Germany) against standard solutions of Mg, Ca, and P with a concentration of 5 and 10 ppm, respectively. X-ray diffraction patterns were recorded on a Siemens D5005 diffractometer (Siemens, Karlsruhe, Germany) in a 2 Theta range from 20–40° and a step size of 0.02°. The diffraction patterns were compared to reference structures of farringtonite (PDF-No. 33-0876), struvite (PDF-No. 15-0762), stanfieldite (PDF-No. 11-0231), α-tricalcium phosphate (PDF-No. 29-0359), monetite (PDF-No. 09-0080), and hydroxyapatite (PDF-No. 09-0432) for qualitative phase analysis.

### 2.3. Animal Experiments

The animal study was approved by local authorities (Regierungspräsidium Unterfranken, No. 55.2 2532-2-338) and was in compliance with international recommendations for care and use of laboratory animals (ARRIVE guidelines and EU Directive 2010/63/EU for animal experiments). 24 New Zealand white rabbits (age 13 weeks) with a weight of approximately 3 kg were used and randomly divided into four groups. The materials were implanted bilateral into the femur of the hind limbs in a non-load-bearing drill-hole defect at the distal femoral condyle. All surgical procedures were performed under general anesthesia by intramuscular injection of ketamine (60 mg kg^−1^ weight) and xylacine (4 mg kg^−1^ weight), followed by inhalation of isofluran (CP-Pharma GmbH, Burgdorf, Germany). The surgical area was shaved and the skin was incised unilaterally and a bore-hole was drilled into the distal lateral epicondyle (d = 5 mm, l = 10 mm). The defects were then flushed with sterile saline to remove bone debris and subsequently filled with the premixed cement pastes. After a setting time of approximately 3–5 min, the defects were closed. The animals were sacrificed after 6 weeks and 12 weeks post-implantation and the cement implants including the surrounding soft tissues were explanted. The femora with adherent muscles were placed into the mag stage of a Bruker Xtreme II imaging system (Bruker Corporation, Billerica, MA, USA) for taking X-ray images. Afterwards the soft tissue was removed and the bones were stored in formaldehyde solution for at least 14 days prior to histological analysis. 

The implant area (condyles and about 10 mm of the femur shaft) were cut from the bone using an Exakt band resaw (Exakt Advanced Technologies GmbH, Norderstedt, Germany). Embedding in Technovit 7200 (Heraeus Kulzer GmbH, Wehrheim, Germany) was performed according to Donath [18]. In short, the samples were dehydrated in an ascending series of Technovit 7100 followed by infiltration with Technovit 7200 in 5 steps, each step lasting 48 h. After embedding and hardening in a mold by UV-light, the samples were mounted on slides as a sandwich and slices were cut with the band resaw. The slices were grinded down to a thickness of about 20 µm. To analyze the implant’s tissue contact, the slices were stained using the Masson–Goldner–Trichrom staining procedure [19]. 

The circumference of the implant in contact with mineralized bone, osteoid, or connective tissue was measured by using the ImageJ software (NIH, Bethesda, MD, USA). Statistical analysis was performed by means of SPSS (IBM SPSS Statistics for Windows, Version 25, Armonk, NY, USA: IBM Cor) applying the independent t-test and the dependent t-test, respectively, depending on whether the implants were in the same animal or not. 

## 3. Results 

**Figure 1:**
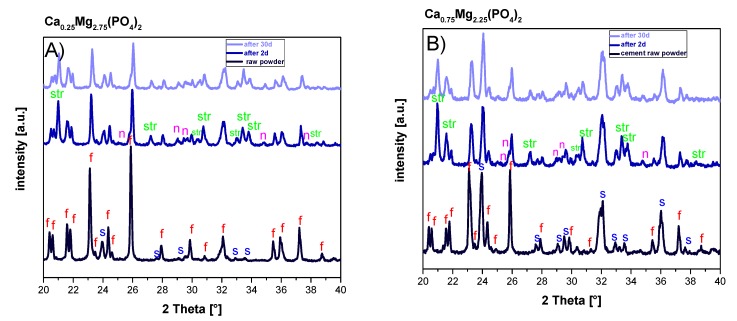

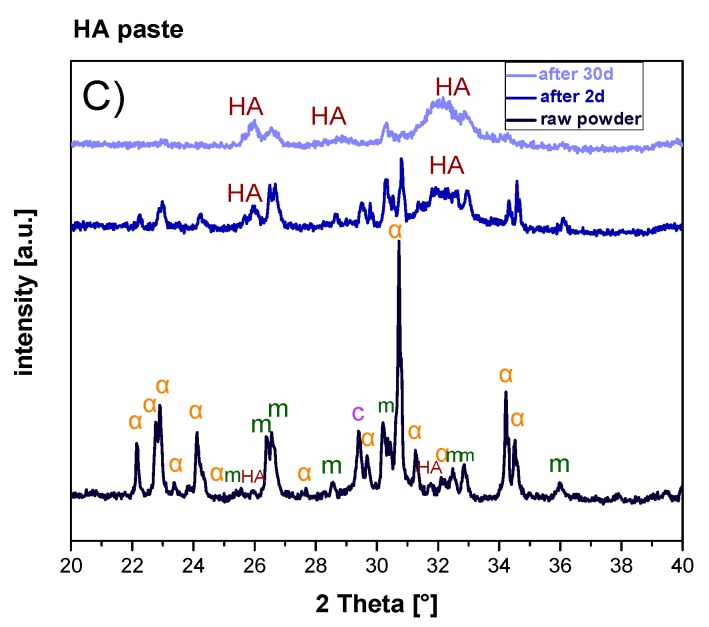
X-ray diffraction patterns of the raw powders as well as premixed cement pastes derived from Ca_0.25_Mg_2.75_(PO_4_)_2_ (**A**) and Ca_0.75_Mg_2.25_(PO_4_)_2_ (**B**) and the HA forming reference cement (**C**) after 2 and 30 d immersion in PBS buffer. Peaks are labelled with: α: α-TCP, m: monetite, c: calcite, HA: hydroxyapatite, f: farringtonite, s: stanfieldite, str: struvite, and n: newberyite.

The setting reaction of the cement pastes was analyzed during immersion in PBS buffer for up to 30 days. According to X-Ray diffraction, the CaMgP raw powders were mainly composed of farringtonite (Mg_3_(PO_4_)_2_) with a minor fraction of stanfieldite (Ca_3_Mg_3_(PO_4_)_4_) (Figure 1). The reaction of the CaMgP powders with the ammonium phosphate in the paste was initiated by mixing with the aqueous solution in the static mixer of the double chamber cartridge and resulted in the predominant formation of struvite (MgNH_4_PO_4_·6H_2_O) and a minor fraction of newberyite (MgHPO_4_·3H_2_O). After 30 days in PBS, residues of farringtonite and stanfieldite were still present in the set CaMgP cements (Figure 1A,B). In contrast, all reflection peaks from the cement raw material of the reference paste (α-TCP, monetite) disappeared over the course of 30 days and only hydroxyapatite could be observed in the set cements (Figure 1C).

**Figure 2:**
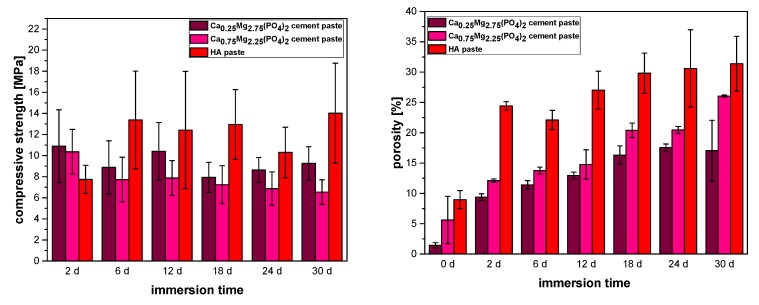
Compressive strength (**left**, n = 9) and porosity **(right**, n = 3) of samples prepared from premixed CaMgP and HA forming cement pastes during incubation in PBS for 30 d at 37 °C.

Figure 2 shows the variation of compressive strength and porosity during immersion in PBS buffer for up to 30 days. For the Ca_0.25_Mg_2.75_(PO_4_)_2_ cement paste, compressive strength decreased from initially 10.8 MPa to 9.3 MPa at the end of the incubation period. At the same time, an increase of porosity from 1.9% to 16.1% could be observed. For the Ca_0.75_Mg_2.25_(PO_4_)_2_ paste similar results were observed, albeit to a greater extent. While porosity increased from 5.5% to 26.1%, compressive strength decreased continuously from 10.4 MPa to 6.5 MPa after 30 days in PBS. In contrast, the reference paste displayed an initial increase of compressive strength from 7.8 MPa (day 2) to 13.4 MPa (day 6) and then no further significant increase (14.0 MPa, day 30). Porosity increased in a similar manner from initially 9.2% to 24. % (day 2), continuing with a slower increase up to 31.1% (day 30).

**Figure 3:**
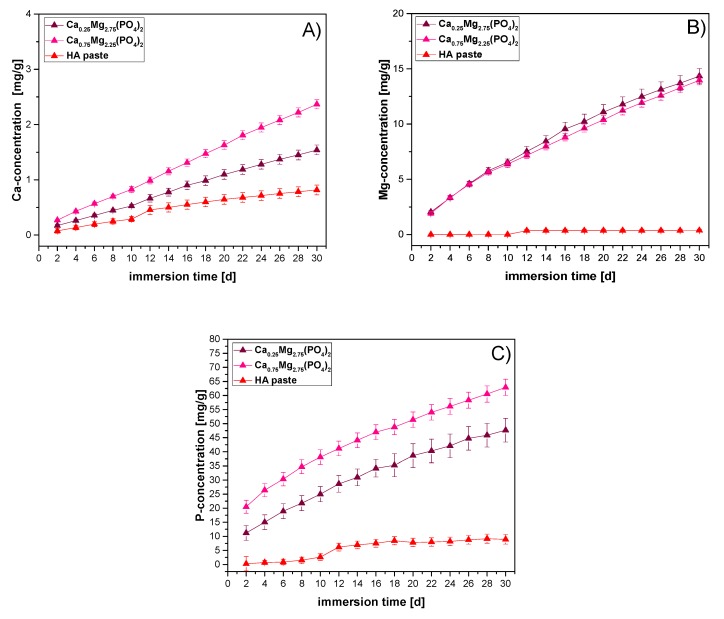
Cumulative calcium- (**A**), magnesium- (**B**) and phosphate release (**C**) from the premixed CaMgP and HA forming cement pastes during incubation in PBS for 30 d at 37 °C. Values are given as mass of dissolved ions related to one gram of set cement.

The material degradation of the cements during immersion in PBS was further studied by measuring the released concentration of Ca^2+^, Mg^2+^ and HPO_4_^2−^ (Figure 3). In general, a constant release of Mg^2+^, Ca^2+^, and HPO_4_^2−^ ions could be observed for all cement pastes at every point of measurement, however, the extent varied. For both CaMgP cement pastes, a 7-fold increase of Ca^2+^- and Mg^2+^-concentration (Figure 3A,B) and a 3-4-fold increase of HPO_4_^2−^ -concentration (Figure 3C) could be detected. For the HA paste, only the Ca^2+^-concentration increased 8.5-fold, whereas Mg^2+^- and HPO_4_^2−^ -concentrations remained nearly zero. After 30 days of storage in PBS, an overall degradation of 6.35 wt.% of the Ca_0.25_Mg_2.75_(PO_4_)_2_ cement, 7.9 wt.% of the Ca_0.75_Mg_2.25_(PO_4_)_2_ cement and 0.97 wt.% of the reference cement was observed (Table 1). 

### Animal Experiments

In general, the tested premixed cement pastes showed good biocompatibility. There was no formation of soft connective tissue observable and no signs of inflammation, like macrophages, could be detected in any sample. 

**Figure 4:**
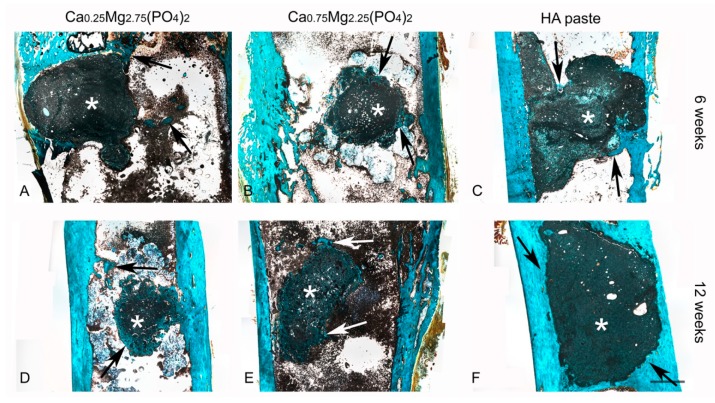
Histological slices of the Ca_0,25_Mg_2,75_(PO_4_)_2_ paste (**A**,**D**), the Ca_0,75_Mg_2,25_(PO_4_)_2_ paste (**B**,**E**) and HA paste (**C**,**F**), 6 and 12 weeks after implantation into drill hole defects at the distal lateral epicondyle of New Zealand white rabbits. The remaining cement pastes appear in dark green and black color (asterisk). Staining colors: keratin, muscle fiber: red; mineralized bone: blue/turquoise; non-mineralized bone: orange/brownish. Arrows indicate areas of newly formed bone. Scale bar = 2 mm.

**Figure 5:**
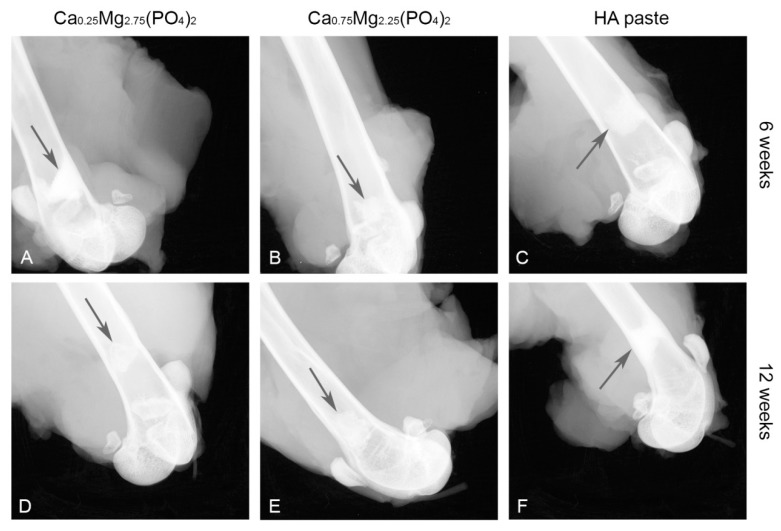
X-ray images of the Ca_0,25_Mg_2,75_(PO_4_)_2_ paste (**A**,**D**), the Ca_0,75_Mg_2,25_(PO_4_)_2_ paste (**B**,**E**) and HA paste (**C**,**F**) in the femoral defect directly after explantation after 6 and 12 weeks. The implants are marked by arrows.

For the HA paste close contact to mineralized bone as well as some osteoid areas could be observed, whereas the volume of the paste remained constant (see Figure 5C,F) over the course of the study (Figure 4C,F). Newly formed mineralized bone was detectable surrounding the cement implants (arrows in Figure 4C,F). On the right side of Figure 4C, a thin bony lamella can be identified, which covered the cement paste and thus closed the drill hole completely after 6 weeks. At 12 weeks the thickness of the compacta was increased. 

Similar findings with respect to osteoid and bone formation were observed for the CaMgP cement pastes. A complete regeneration of the cortical bone could be observed after 12 weeks for the Ca_0.25_Mg_2.75_(PO_4_)_2_ cement (Figure 4A,D) as well as for the Ca_0.75_Mg_2.25_(PO_4_)_2_ cement (Figure 4B,E). Furthermore, after 6 weeks there was a thin non-calcified osteoid-lamella detectable for the Ca_0.25_Mg_2.75_(PO_4_)_2_ cement, which covered the implant surface (left side, Figure 4B). At both time points newly formed bone was also visible for both formulations. In general, the rim of the cement samples was more fissured for the Mg-containing samples compared to the HA paste.

X-ray imaging revealed, that the HA paste showed no difference in X-ray opacity when comparing 6 weeks and 12 weeks post-implantation (Figure 5C,F). In contrast, X-ray opacity decreased for the Ca_0,25_Mg_2,75_(PO_4_)_2_ paste (Figure 5A,D) and also for the Ca_0,75_Mg_2,25_(PO_4_)_2_ paste (Figure 5B,E). 

**Figure 6:**
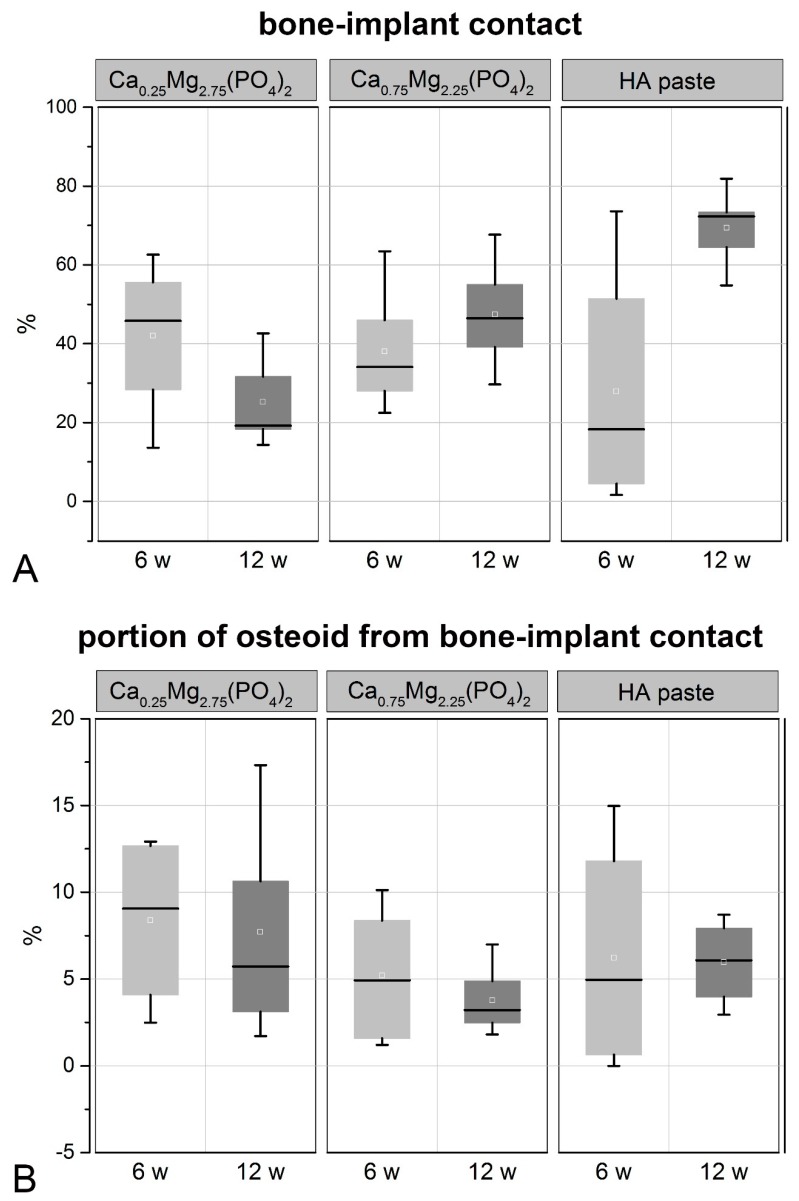
Bone-implant contact (**A**) and the portion of osteoid from bone-implant contact (**B**) determined from histological sections 6 and 12 weeks after implantation, respectively.

The analysis of the histological images with respect to the bone-implant-contact revealed the highest bone-implant contact after 12 weeks for the HA paste, which was significantly higher than for the CaMgP pastes (Figure 6A). On the contrary, the implant contact after 6 weeks was higher for CaMgP samples compared to the HA paste and was highest for the Ca_0.25_Mg_2.75_(PO_4_)_2_ paste. The bone-implant contact area further increased from 6 to 12 weeks for the Ca_0.75_Mg_2.25_(PO_4_)_2_ and the HA pastes. In contrast, a decrease of bone-implant contact was observed for the Ca_0.25_Mg_2.75_(PO_4_)_2_ paste after 12 weeks. After this time span the bone-implant contact of the Ca_0.75_Mg_2.25_(PO_4_)_2_ paste was significantly higher than for the Ca_0.25_Mg_2.75_(PO_4_)_2_ paste. 

The percentage of osteoid was not significantly different between all groups analyzed. However, the osteoid percentage was slightly higher for the Ca_0.25_Mg_2.75_(PO_4_)_2_ paste compared to the Ca_0.75_Mg_2.25_(PO_4_)_2_ and the HA pastes after 6 weeks. (Figure 6B). The osteoid percentage then decreased from 6 to 12 weeks for the CaMgP pastes, whereas it slightly increased for the HA paste.

## 4. Discussion

Magnesium substitution in CaP cements as well as pure MPC has captured increasing attention in bone graft research during the last few years [8]. This material group exhibits several features which are relevant for the application in clinical situations. Examples are the intrinsic anti-microbial effects of MgPC which may be beneficial in osteomyelitis patients [20], their use as bone adhesive [21] as well as materials for maxillary sinus grafting [22]. Therefore, introducing magnesium ions into CaP-bone cements may improve the clinical outcome.

Premixed mineral bone cements offer superior handling properties compared to conventional liquid-powder approaches during application, while maintaining their unique properties and suitability for repair, augmentation, and regeneration of bone defects. In this context, the successful development of an injectable ready-to-use calcium phosphate cement formulation using water-immiscible carrier liquids has been demonstrated previously [9]. Based on this technology, the current study describes the characterization of premixed calcium doped magnesium phosphate cement pastes with improved degradation properties under in vitro and in vivo conditions. 

The CaMgP cement pastes were applied using a double chamber syringe system, where an aqueous solution of potassium phosphate was used as initiator of the cement reaction. This 2-component approach was chosen to ensure flexible handling and fast working times during implantation. Mixing of both components occurred by extrusion through a static mixer and resulted in a rapid hardening of the cements within several minutes. During setting, the reaction of CaMgP raw powders, consisting of the mineral phases farringtonite and stanfieldite, with the admixed ammonium phosphate led to the formation of struvite [1]. The ratio of the cement raw powder to the phosphate salts in the overall mixture was not stoichiometric and therefore residues of farringtonite and stanfieldite were still present in the set cements after prolonged storage times in PBS. In contrast, the calcium phosphate cement paste used as a reference material continuously converted into nanocrystalline hydroxyapatite.

In the described CaMgP cements, the oil phase within the pastes acted as a pore generator leading to initially quite low porosities <10 %, which subsequently increased to values of 16–25% after 30 days in PBS. In contrast, a higher porosity for the HA forming reference paste is due to the higher density of the setting product hydroxyapatite (d ~ 3.1 g ml^−1^) compared to the struvite (d ~ 1.7 g ml^−1^) and newberyite (d ~ 2.1 g ml^−1^) formed within the CaMgP cements [23,24]. In addition, both magnesium phases are highly hydrated and most likely consumed most water from the surrounding solution resulting in initially low porosity values. This is in accordance with water-based struvite cement pastes, which also show porosity values of 5–7% directly after setting [25]. The mechanical stability of the CaMgP pastes was found to be in a range of 7–10 MPa under compressive load with only a slight decrease over time. The strength of the reference cement initially increased from 8 MPa after 2 days to ~13–14 MPa, which can be attributed to the proceeding setting reaction. The compressive strength then remained on this level until the end of the experiment, despite of the continuously increasing porosity. This further demonstrates that the strong increase in porosity for all cement pastes must be related to the release of the oil phase and not material degradation, since the latter would normally result in a massive loss of mechanical stability [26]. Generally, the mechanical strength of the premixed cements from our study is lower than the strength of injectable aqueous cement pastes for both CPC (approximately 2 MPa up to 83 MPa depending on their preparation and treatment during setting [27,28]) and MPC (~66 MPa for similar compositions as in this study [1,29]). The reason for this different behavior is likely related to the higher porosity of the oil-cement pastes, e.g., the porosity of aqueous struvite cements was reported to be only 5–7% after setting [25] compared to ~20–25% porosity of the oil-cement pastes from this study after oil release. In addition, crystal growth in an oil-surfactant-water mixture might have resulted in a worse interlocking of the precipitated cement crystals compared to pure aqueous cement pastes. 

To assess the degradation potential of the CaMgP cement pastes, the ion release of Mg^2+^, Ca^2+^, and HPO_4_^2−^ was examined by means of ICP-MS. The release of Ca^2+^ was found to be approximately one order of magnitude lower than the release of HPO_4_^2−^ or Mg^2+^. This is in accordance with results previously reported for comparable struvite-based cements and can be attributed to the small Ca:Mg ratio for such materials [30]. Furthermore, the release kinetics of Mg^2+^ and HPO_4_^2−^ obviously followed a zero order kinetic with a constant ion release of ~0.5 mg/g per day. At the end of the experiment (30 d), roughly 7–8% of the cements were dissolved, whereas dissolution appeared to be incongruent as much less calcium was released during this time. In contrast, a much lower ion release was observed for the HA paste resulting in a total mass loss of ~0.98%, confirming the lower solubility of calcium phosphate-based bone cements.

In vivo, both CaMgP cement pastes induced vital bone formation according to the results of the histological analysis of the implants. A fast initial ingrowth of vivid bone cells into the medullary cavity resulted in a complete regeneration of the femur corticalis within the drill hole region after 12 weeks. Similar results have already been presented for MPCs in an ovine femoral defect model, where struvite cements displayed almost complete degradation after 10 months accompanied by new bone formation [7]. MPCs have also been subject to studies in rodents, where 3D printed scaffolds were implanted in a rat calvaria defect model leading to enhanced bone regeneration, even with partially increased bone volume [31]. During the bone regeneration process, the CaMgP cement pastes in the current study were completely surrounded by newly formed bone tissue, whereas the initial bone-implant contact (6 weeks) was higher compared to the HA paste. Furthermore, the implants gradually decreased in size until the end of the experiment (12 weeks), most likely due to resorption processes, which may also explain the more fissured surface of the samples compared to the reference paste. In addition, decrease of X-ray opacity supported the finding that the CaMgP cements were successively degraded in vivo. Radiographic changes indicating a faster and more distinct dissolution of MPCs compared to CaP based cements in heterotopic rodent models have already been reported previously [32]. While the bone-implant contact was increasing for Ca_0.75_Mg_2.25_(PO_4_)_2_ from 6 to 12 weeks, the opposite was observed for the Ca_0.25_Mg_2.75_(PO_4_)_2_ paste, which might be related to the fast degradation of the material. In this case, bone ingrowth might not have been able to keep up with the speed of material degradation and close bone- or osteoid-implant contact couldn’t be achieved. On the other hand, a slightly higher osteoid-percentage observed for Ca_0.25_Mg_2.75_(PO_4_)_2_ may indicate a better stimulation of bone growth compared to the Ca_0.75_Mg_2.25_(PO_4_)_2_ paste. The HA paste showed similar osseointegration as the CaMgP materials with good bone-implant contact. However, as expected, only marginal degradation of the paste was observed after 6 and 12 weeks, which is in accordance with results reported in the literature [33]. Additionally, this was supported by X-ray imaging, which revealed practically no change in X-ray opacity during this time period, indicating the absence of any resorption effects. 

It should be noted that using individual animals may lead to runaway values due to individual reactions to the implants. Animal models are useful to obtain a first hint of the functionality of newly developed implant materials. Especially the rabbit model is well established due to the easy handling and size of the animals making them an ideal model for screening materials in preliminary studies before using large animals for selected materials. However, one has to keep in mind that the faster bone metabolism is associated with faster healing compared to large animals and humans [16,17]. Nevertheless, in the rabbit model the outcome of bony integration of different materials can be compared and promising formulations can be identified and analyzed further.

In summary, the different calcium doping had a moderate influence on the material properties and biological performance of the CaMgP pastes. Both cement paste formulations mainly led to the formation of struvite and newberyite, whereas the ion-release into PBS under in vitro conditions was comparable. However, CaMgP cement paste with low calcium content showed higher mechanical stability in terms of compressive strength. Regarding the promotion of bone regeneration in vivo, both CaMgP pastes supported proper bone ingrowth, albeit a low calcium content appears to cause faster resorption and higher osteoid percentage according to histological and radiographic findings. On the other hand, bone-implant contact was found to be higher for the CaMgP paste with high calcium content at the end of the implantation study, which can most likely be attributed to a delayed resorption.

## 5. Conclusions

Premixed oil-based bone cement pastes with CaMgP chemistry combine the advantages of conventional MPCs such as high mechanical strength and good resorbability with a prolonged shelf-life and easy handling during clinical application. By dispersing calcium doped magnesium phosphate cement powders of different stoichiometric Mg:Ca ratios and ammonium phosphate in an oil-surfactant mixture, it was possible to produce reactive cement pastes with good consistency. By mixing such pastes with an aqueous solution of potassium phosphate, we were able to create cements predominantly consisting of pure struvite and newberyite and displaying appropriate compressive strength as well as degradability in PBS under *in vitro* conditions. Animal experiments revealed excellent results in bone healing with complete osseointegration of the CaMgP pastes into the bone defects and successive resorption of the materials depending on the calcium content. In this context, such materials may offer the possibility to adapt the degradation rate of the cements to the bone regeneration capacity. In summary, the CaMgP cement pastes presented herein are a promising alternative to conventional calcium phosphate based bone replacement materials, due to their excellent material properties with respect to biocompatibility and degradation as well as ease of application.

## Figures and Tables

**Table 1:** Cumulative release of calcium, magnesium and phosphate from samples prepared from premixed CaMgP cement pastes after 30 d immersion in PBS at 37 °C.

	Ca_0.25_Mg_2.75_(PO_4_)_2_	Ca_0.75_Mg_2.25_(PO_4_)_2_	HA Paste
**calcium**	0.15 wt.%	0.2 wt.%	0.08 wt.%
**magnesium**	1.4 wt.%	1.4 wt.%	-
**phosphate**	4.8 wt%	6.3 wt.%	0.89 wt.%
